# Immune fitness and biomarkers of immune function: Relationships with the oral and gut microbiome composition

**DOI:** 10.1016/j.bbih.2026.101239

**Published:** 2026-04-14

**Authors:** Guusje A. Ulijn, Emina Išerić, Aurora J.A.E. van de Loo, Johan Garssen, Phillip A. Engen, Ankur Naqib, Stefan J. Green, Ali Keshavarzian, Joris C. Verster

**Affiliations:** a Utrecht University, Division of Pharmacology, Utrecht Institute for Pharmaceutical Sciences, Universiteitsweg 99, 3584 CG, Utrecht, the Netherlands; b Danone Global Research and Innovation Center, Uppsalalaan 12, 3584CT, Utrecht, the Netherlands; c Rush Center for Integrated Microbiome and Chronobiology Research, Rush University Medical Center, Chicago, IL, USA; d Rush Research Bioinformatics Core, Rush University Medical Center, Chicago, IL, USA; e Genomics and Microbiome Core Facility, Rush University Medical Center, Chicago, IL, USA; f Department of Internal Medicine, Rush University Medical Center, Chicago, IL, USA; g Department of Anatomy and Cell Biology, Rush University Medical Center, Chicago, IL, USA; h Department of Physiology, Rush University Medical Center, Chicago, IL, USA; i Cognitive Neurophysiology, Department of Child and Adolescent Psychiatry, Faculty of Medicine, TU Dresden, D-01307, Dresden, Germany; j Centre for Mental Health and Brain Sciences, Swinburne University, Melbourne, VIC, 3122, Australia

**Keywords:** Immune fitness, Immunity, Microbiota, Inflammation biomarkers, Saliva, Stool

## Abstract

**Background:**

Immune fitness refers to the body's capacity to respond to health challenges, such as infections, by activating an appropriate immune response. The aim of the current study was to investigate the relationship between oral and gut microbiota community structure and immune fitness scores.

**Methods:**

Stool and saliva samples were collected to assess compositions of both oral and gut microbiota. Immune fitness was assessed with a single-item scale ranging from 0 (very poor) to 10 (excellent). Additionally, saliva samples were analyzed to measure the concentrations (pg/ml) of pro-inflammatory biomarkers interleukin (IL)-1β, IL-6, IL-8, and tumor necrosis factor-alpha (TNF-α). Spearman's correlations were computed between microbiota abundance, immune fitness, and salivary biomarker levels. Bootstrapping was used to adjust for the relatively small sample size in the correlation analysis.

**Results:**

A total of 29 healthy participants (15 males; 14 females) enrolled in the study, with a mean age of 21.1 years old. Analysis of the salivary microbiota revealed significant negative correlations between self-reported immune fitness scores and the relative abundances of putative oral proinflammatory genera *Selenomonas* (r = −0.610)*,* and *Lachnospiraceae* uncultured (r = −0.501). In the fecal microbiota, immune fitness scores showed a significant positive correlation with the relative abundance of putative beneficial butyrate-producing genus *Lachnoclostridium* (r = 0.513), and significant negative correlations with commensal gut bacterial genera *Colidextribacter* (r = −0.582) and *Lachnospiraceae FCS020* group (r = −0.504).

**Conclusion:**

Self-reported immune fitness is associated with the oral and gut microbiota community. The findings demonstrate the importance of microbiota in immune function and support the use of self-assessment scales to evaluate immune fitness.

## Highlights

• Immune fitness is significantly correlated with the oral and gut microbiota.
• Negatively with salivary *Selenomonas* and *Lachnospiraceae* uncultured.
• Positively with fecal *Lachnoclostridium*.
• Negatively wit fecal *Colidextribacter* and *Lachnospiraceae FCS020* group.

## 1 Introduction

Immune fitness is defined as the capacity of the body to respond to health challenges, such as infections, by activating an appropriate immune response, which is essential to maintain health (Verster et al., 2022, 2023a). Immune fitness can be determined using a single-item patient-reported outcome measure in which the patient is asked to rate their immune fitness on a scale from 0 to 10 (Verster et al., 2022). Although self-assessed immune fitness is a subjective measure, it has been found to correlate significantly with mood disorders (e.g. depression), disease states, and overall quality of life (Verster et al., 2022; Išerić et al., 2026). Experiencing reduced immune fitness could stimulate a person to take appropriate action, such as consulting a medical professional or making lifestyle changes in order to improve their immune fitness (Verster et al., 2022). These forms of action, for example altering dietary habits or increasing the level of physical activity, could potentially improve the immune fitness and overall health of patients (Išerić et al., 2026).

Host-associated microbiomes are dynamic communities of microorganisms consisting of bacteria, fungi, viruses, archaea, and their genes that colonize the human host (Hou et al., 2022). An ideal microbiome promotes both organ and systemic health due to the overall symbiotic and dynamic relationship with its host (Kuziel and Rakoff-Nahoum, 2022; Huang et al., 2023). Emerging evidence strongly suggests that commensal microbiota significantly impact human health and disease primarily through their roles in immune and metabolic regulation (Hou et al., 2022). Host-associated microbiota can mediate the impact of environmental and lifestyle factors, such as daily diet, stress and exercise, on the host organ and systemic immunity (Belkaid and Hand, 2014; Veldhoen and Brucklacher-Waldert, 2012; Sohail et al., 2019).

A relationship exists between the composition of the salivary microbiome and the development of oral diseases, as well as its association with systemic diseases, autoimmune disorders and various types of cancer (Baker et al., 2024; Rathee and Gingivitis, 2023). Notably, salivary bacterial genera *Streptococcus* and *Fusobacterium* have been found to contribute to the development of gingivitis (Zilberstein et al., 2025). Additionally, a bidirectional relationship between periodontal disease and gut inflammation was found in inflammatory bowel disease (IBD) patients, emphasizing the role of oral and gut microbiome imbalances in driving immune dysregulation (Qi et al., 2021a). Patients with early rheumatoid arthritis showed an increased abundance of the genera *Veillonella* and *Prevotella* in their saliva compared to healthy controls (Kroese et al., 2021). Consequently, the composition of the oral microbiome in saliva is strongly associated with both systemic and oral health, particularly in relation to immune-related health complaints. Similar relationships have also been seen between immune-related complaints and the gut microbiome (Raygoza Garay et al., 2023; Liu et al., 2013).

Pro-inflammatory cytokines such as interleukin (IL)-1β, IL-6, IL-8 and tumor necrosis factor (TNF)-α are released by immune cells to induce local or systemic inflammation in the human body (Diesch et al., 2021). A significant increase in levels of these pro-inflammatory cytokines are therefore signs of systemic inflammation, that could indicate an underlying infection or disease (Hirano, 2021). For example, IL-6, IL-8 and TNF-α have been found in elevated levels in saliva of patients with dental caries disease (Gornowicz et al., 2012) and IL-1β and IL-8 levels were elevated in patients with joint and muscle diseases (Rathnayake et al., 2013). Also, a strong correlation between increased pro-inflammatory cytokine levels and cancer development has been found (Singh et al., 2019). Alterations in cytokine levels have been linked to specific microbial compositions in both the oral and gut microbiomes. For example, the relative abundance of *Bifidobacterium adolescentis* and *Haemophilus spp.* in the gut have been negatively correlated with blood TNF-α levels (Schirmer et al., 2016; Wang et al., 2021). Conversely, in obese patients, the relative abundance of gut bacteria from the genus *Lactobacillus* has been positively correlated with IL-6 levels (Cooper et al., 2016). These findings underscore the clear link between microbiome and immune function.

The aim of the current study was to evaluate the associations between perceived immune function, i.e. immune fitness, and microbiome composition. To this end, the relative abundance of microbial taxa in both the oral (saliva) and gut (stool) samples were interrogated in a cohort of young, healthy volunteers. Additionally, concentrations of pro-inflammatory cytokines IL-1β, IL-6, IL-8 and TNF-α were measured. It was hypothesized that microbial taxa involved in immune regulation would be significantly associated with self-reported immune fitness.

## 2 Methods

### 2.1 Study participants

This study utilized data from 29 healthy participants collected from the control day (no intervention) of a larger investigation into the effects of alcohol hangover (Mackus et al., 2023). The University of Groningen Psychology Ethics Committee approved the study (protocol code: ppo-015–002, approval date: 3 September 2015). Male and female social drinkers (18-30 years old) were recruited as participants through local advertisement, gave written informed consent before start of the study, and received €120,- for their participation. Potential participants were screened at which exclusion criteria were evaluated, including smoking and drug use, having an underlying disease or using medication. No diet instructions were given to the participants. Participants were excluded if they had a poor night of sleep prior to the test day or experienced minor illness on the test day. The participants’ health status and medication use were evaluated by the study physician at the start of the test day. All participants were tested for the absence of recent use of alcohol, by using the Alcotest 7410 Breath Alcoholmeter (Dräger, Hoogvliet, the Netherlands). To verify the use of illicit drugs (including amphetamines, barbiturates, cannabinoids, benzodiazepines, cocaine, and opiates) a urine drug test was conducted (AlfaScientic Designs Inc., Poway, CA, USA).

On the test day, no food and drinks were allowed after waking up, and when they arrived at the Institute, all participants received a standard breakfast (a curran bin and glass of water or milk) at 09.00 a.m. All 29 participants completed the immune fitness scale at 09:30 a.m. and at the same time a saliva sample was collected. For a sub-set of 16 participants, a stool sample was collected between 09:30 and 15:30, at a moment that was convenient for the participant. Immune fitness was assessed hourly from 09:30 to 15:30 with an 11-point single-item rating scale ranging from 0 (very poor) to 10 (excellent) (Verster et al., 2022). The single-item rating has been validated against the multiple-item Immune Status Questionnaire (ISQ), and its outcome correlates significantly with various lifestyle and health outcomes (Verster et al., 2022), and biomarkers of immune function such as IL-6 (Išerić et al., 2025a). The single-item immune fitness ratings showed to be stable across seasons (Išerić et al., 2025b), and have a high test-retest reliability (Verster et al., 2023b).

### 2.2 Saliva and stool collections

The saliva samples were collected at 09:30 a.m. using a passive drool method (SalivaBio's Saliva Collection Aid, Salimetrics, State College, PA, USA). Stool was collected at variable time points during the test day, depending on the participants' need to defecate. Both saliva and stool samples were stored at −80 °C until experimental analysis.

### 2.3 Saliva cytokine determination

Applying standard procedures for saliva processing described elsewhere (Van de Loo et al., 2021), saliva concentrations (pg/ml) of interleukin (IL)-1β, IL-2, IL-4, IL-5, IL-6, IL-8, IL-10, granulocyte–macrophage colony-stimulating factor (GM-CSF), interferon-gamma (IFN-γ) and tumor necrosis factor-alpha (TNF-α) were determined in duplicate via multiplex immunoassay (customized ProcartaPlex Immunoassay, ThermoFisher Scientific, Waltham, USA) with a Luminex LX 200 (Luminex corp., Diasorin, Austin, Texas USA). The incubations were conducted at room temperature. For each sample, 25 μL was transferred to a dilution plate, and 25 μL of assay buffer was added. Magnetic beads were prepared by dilution with 4 × buffer and pipetted into a 96-well plate. After washing the beads twice, standards and the samples were added to the plate (30 min incubation time). The plate was washed twice and diluted biotinylated detection antibody was added, followed by an incubation time of 30 min. Then, the plate was washed twice and diluted, streptavidin– phycoerythrin added as detection substrate, and incubated for 10 min. The plates were washed twice and assay buffer was added to each well. Fluorescence was read within 30 min. For each multiplex plate, the lower limit of detection (LOD) was computed, and for concentrations below the LOD, half the LOD value was used for the statistical analyses. If more than 25 % of the biomarker assessments were below LOD, the assessment was considered unreliable and the biomarker was excluded from further analysis (Van de Loo et al., 2021). Reliable biomarker data was obtained for IL-1β, IL-6, IL-8, and TNF-α.

### 2.4 DNA extraction

Total DNA was extracted from human saliva and stool samples using a FastDNA bead-beating Spin Kit for Soil (MP Biomedicals, Solon, OH, USA), and verified with fluorometric quantitation (Qubit, Life Technologies, Grand Island, NY). The extracted genomic DNA was subsequently PCR-amplified using a two-stage protocol as described in prior works (Naqib et al., 2018; Caporaso et al., 2012), and employing the Earth Microbiome Project (EMP) validated universal primers 515F/806R (515F:GTGYCAGCMGCCGCGGTAA; 806R:GGACTACNVGGGTWTCTAAT) targeting variable region four (V4) of microbial 16S ribosomal RNA (rRNA) genes. Negative controls were used with each set of amplifications, which indicated no contamination. This EMP primer set provided broad phylogenetic coverage and is widely used in microbiome studies.

Amplicon libraries were pooled in equal volumes using an epMotion 5075 automated liquid handling system (Eppendorf, Hamburg, Germany). The pooled library was purified using AMPure XP magnetic beads (Beckman Coulter, cat. no. A63881) at a 0.6 × (vol/vol) bead-to-sample ratio to remove primer dimers and short nonspecific fragments. Library fragment size distribution was assessed prior to sequencing to confirm the expected V4 amplicon length (∼292–293 bp).

Purified libraries were spiked with 20% PhiX control DNA (Illumina, cat. no. FC-110-3001) to increase sequence diversity and loaded onto an Illumina MiSeq instrument using a MiSeq Reagent Kit v2 (300-cycle; Illumina, cat. no. MS-102-2002), generating paired-end 2 × 150 bp reads. The selected read configuration provides sufficient overlap to reliably merge paired-end reads spanning the full V4 region. Following the initial sequencing run, read counts per barcode were evaluated, and individual amplicon libraries were re-pooled prior to purification to achieve a more balanced distribution of sequencing depth across samples. The re-pooled and re-purified libraries were subsequently sequenced on a second MiSeq run using identical sequencing parameters.

Library preparation, pooling, and MiSeq sequencing were performed at the Argonne National Laboratory.

### 2.5 Microbiome bioinformatics

Raw sequence data were processed using the QIIME2 software package (version 2023.5) (Bolyen et al., 2019). Sequences were checked for quality with FastQC and merged using PEAR (Zhang et al., 2014). The merged sequences were quality-filtered using the q2-demux plugin, followed by denoising with DADA2 (via q2-dada2) (Callahan et al., 2016). Primer adapter sequences were then removed using the cutadapt algorithm (Kechin et al., 2017). Taxonomy was assigned using the q2-feature-classifier classify-sklearn naive Bayes taxonomy classifier against the SILVA 138 99% reference database (Bokulich et al., 2018; Quast et al., 2013). The decontam software identified no contaminants based on the prevalence of amplicon sequence variants (ASVs) in reagent negative blank controls using default settings (Davis et al., 2018). Although no contaminants were detected, host-associated taxa such as mitochondria, chloroplasts, and eukaryote were excluded. Datasets were rarefied to a depth of 43,500 for saliva and 31,500 for fecal samples prior to conducting alpha-diversity analyses (Gihring et al., 2012).

Alpha-diversity metrics—including the Shannon index, Simpson's index, observed features (richness), and Pielou's evenness—and Beta-diversity metrics were computed using q2-diversity within the QIIME environment. Microbial features with relative abundances below 0.1% were ignored for correlation analyses. The relative abundances of the bacterial phyla Firmicutes and Bacteroidetes were calculated from the taxonomically classified 16S rRNA gene sequencing data. For each sample, the Firmicutes-to-Bacteroidetes (F/B) ratio was calculated by dividing the relative abundance of Firmicutes by that of Bacteroidetes.

### 2.6 Statistical analyses

Statistical analyses were conducted with SPSS (IBM Corp. Released, 2013. IBM SPSS Statistics for Windows, Version 30. Armonk, NY, USA: IBM Corp.). Sex differences were determined with the Wilcoxon-signed rank test (p < 0.05 for significance). Spearman's correlations between salivary microbiome outcome measures and the immune fitness and salivary biomarker assessments at 09:30 were computed. To account for the relatively small sample size, bootstrapping was applied (B = 10,000 samples), and the bias-corrected and accelerated 95% confidence interval (BCa 95% CI_B_) was computed (Efron and Tibshirani, 1993; Sideridis and Simos, 2010). The BCa 95% CI_B_ can range from −1 to +1, with a narrower BCa 95% CI_B_ implying a greater precision. For the stool samples, microbiome outcomes were correlated with the 09:30 a.m. immune fitness assessment. Although stool samples were collected at different times during the day, the comparison with the 09:30 a.m. immune fitness rating was deemed warranted, as analyses presented elsewhere revealed that the immune fitness ratings that were performed hourly from 09:30 to 15:30 remained unchanged during the day (Išerić et al., 2025c). Again, bootstrapping was applied to account for the small sample size. Correlations were considered statistically significant *and* clinically relevant if each of the following 3 criteria was met: (a) the magnitude of the Spearman's correlation (r) was greater than ± 0.5, (b) the *p*-value was smaller than 0.05, and (c) the BCa 95% CI_B_ bootstrapping interval did not contain the value zero.

## 3 Results

A total of 29 subjects (15 males and 14 females) participated in the study. Participants were all healthy young adults, without any known disease or registered medication use. Their main demographic characteristics are summarized in Table 1.

**Table 1:** Participant characteristics.

	Overall	Male	Female	*p*-value
Total Number	29	15	14	
Age, in years	21.1 (2.0)	21.5 (1.9)	20.7 (2.1)	0.290
Weight, in kg	72.9 (10.6)	78.8 (10.4)	66.6 (6.5)	0.001∗
Height, in m	1.78 (0.08)	1.84 (0.06)	1.71 (0.04)	<0.001 ∗
BMI, in kg/m^2^	22.9 (2.1)	23.1 (2.5)	22.7 (1.6)	0.715

Mean and standard deviation between brackets are shown. Significant sex differences (*p* < 0.05), determined with the Wilcoxon-signed rank test, are indicated by ∗. Abbreviation: BMI = body mass index.

### 3.1 The oral microbiome

**Fig. 1:**
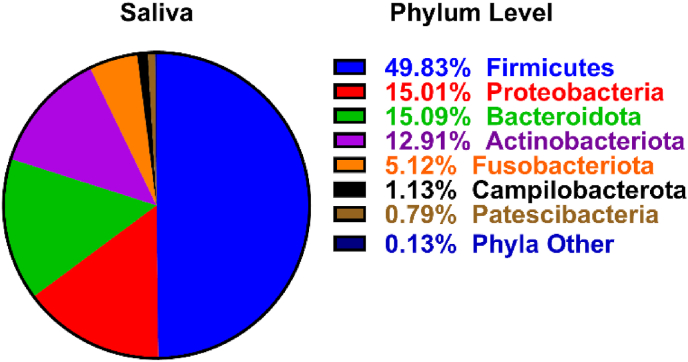
The percentage distribution and relative abundance of salivary bacterial phyla across samples.

**Fig. 2:**
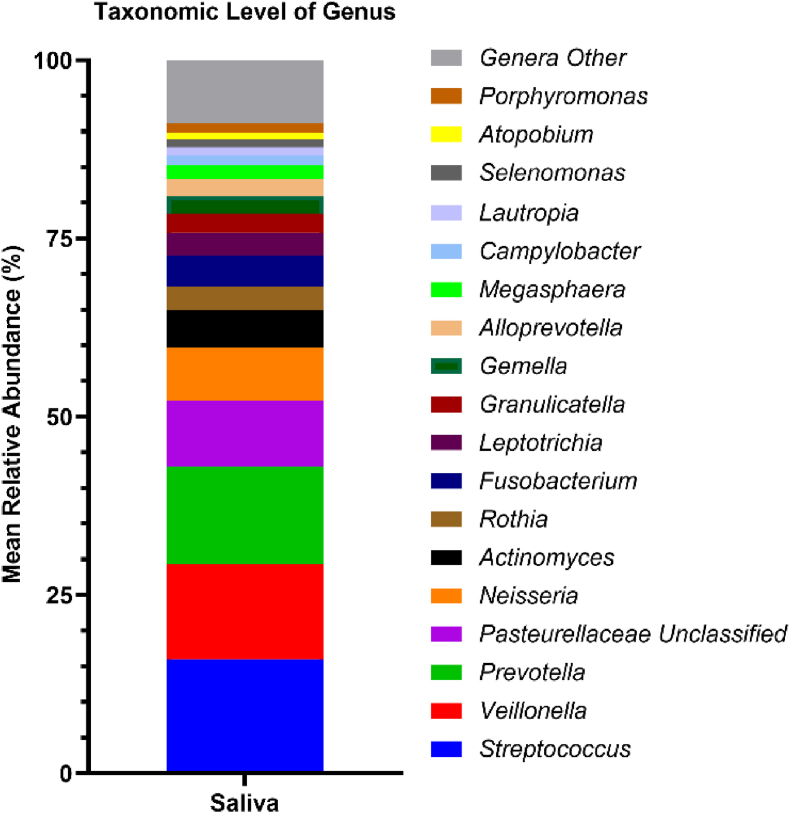
The percentage distribution and relative abundance (>1%) of salivary bacterial genera across samples.

Immune fitness ratings and saliva samples were collected at 09:30 a.m. from all 29 participants. Prior to conducting correlations between saliva microbiota and immune fitness scores and salivary inflammation biomarkers, salivary microbial profiles were assessed at both the phylum (Fig. 1) and genus (Fig. 2) taxonomic levels to visually characterize the bacterial compositions of saliva samples from the participants. As summarized in Fig. 2, the relative abundances of bacterial genera in the saliva, with the most prevalent taxa, included *Streptococcus*, *Veillonella*, *Prevotella*, *Pateurellaceae* Unclassified, and *Neisseria*. These bacterial profiles were derived from a curated set of bacterial genera, ranked by their overall relative abundance (%), and then analyzed using Spearman correlation to assess associations with immune fitness scores and salivary inflammation biomarkers. Additionally, alpha-diversity metrics and Firmicutes/Bacteroidetes (F/B) ratios from the participant saliva samples were included in the correlation analysis, as shown in Table A1, which indicated no significant associations following bootstrapping.

The relationship between the relative genus-level abundances and immune fitness scores are summarized in Table A2. Statistically significant negative correlations were found between immune fitness scores and the relative abundances of putative oral proinflammatory genera *Selenomonas* (r = −0.610; *p* < 0.001; BCa 95% CI_B_ = −0.832, −0.240) and *Lachnospiraceae* uncultured (r = −0.501; *p* = 0.006; BCa 95% CI_B_ = −0.753, −0.145) (Kim et al., 2022; Al-Kamel et al., 2019).

Next, salivary inflammation biomarker levels and bacterial genera associations were examined**.** Table A3 presents the correlations between relative genus-level abundances and salivary cytokine concentrations. Regarding the correlation outcomes between the relative genus-level abundances and salivary IL-1β concentrations, a statistically significant negative correlation was found with the oral anti-inflammatory genus *Lautropia* (r = −0.591; *p* < 0.001; BCa 95% CI_B_ = −0.853, −0.189). A positive correlation with putative oral proinflammatory genus *Saccharimonadales* was observed, but it did not reach bootstrap significance (r = 0.409; *p* = 0.031; BCa 95% CI_B_ = 0.053, 0.686).

Regarding salivary IL-8 concentration, similar to IL-1β, a significant negative correlation was found with the oral anti-inflammatory genus *Lautropia* (r = −0.546; *p* = 0.005; BCa 95% CI_B_ = −0.845, −0.078). A negative correlation with commensal oral genus *Pasteurellaceae* unclassified was noted but did not reach the criteria for significance (r = −0.435; *p* = 0.030; BCa 95% CI_B_ = −0.720, −0.047).

For IL-6, no statistically significant correlations were found due to stringent bootstrapping. However, notable negative correlations were observed between salivary IL-6 and salivary genera *Alloprevotella* (r = −0.386; *p* = 0.042; BCa 95% CI_B_ = −0.698, −0.008), *Bergeyella* (r = −0.408; *p* = 0.031; BCa 95% CI_B_ = −0.684, −0.041), and *Lautropia* (r = −0.427; *p* = 0.024; BCa 95% CI_B_ = −0.723, −0.040).

Finally, for salivary TNF-α concentration also no statistically significant correlations were found due to stringent bootstrapping. However, two notable trends emerged, indicating a negative correlation with the oral anti-inflammatory genus *Lautropia* (r = −0.411; *p* = 0.027; BCa 95% CI_B_ = −0.716, −0.036), like with IL-1β and IL-8, while a positive correlation was observed between TNF-α and the oral opportunistic pathogen genus *Granulicatella* (r = 0.434; *p* = 0.019; BCa 95% CI_B_ = 0.103, 0.703).

### 3.2 Gut microbiome

**Fig. 3:**
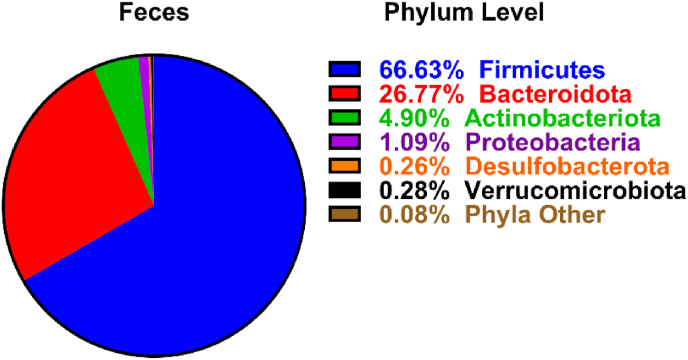
The percentage distribution and relative abundance of fecal bacterial phyla across samples.

**Fig. 4:**
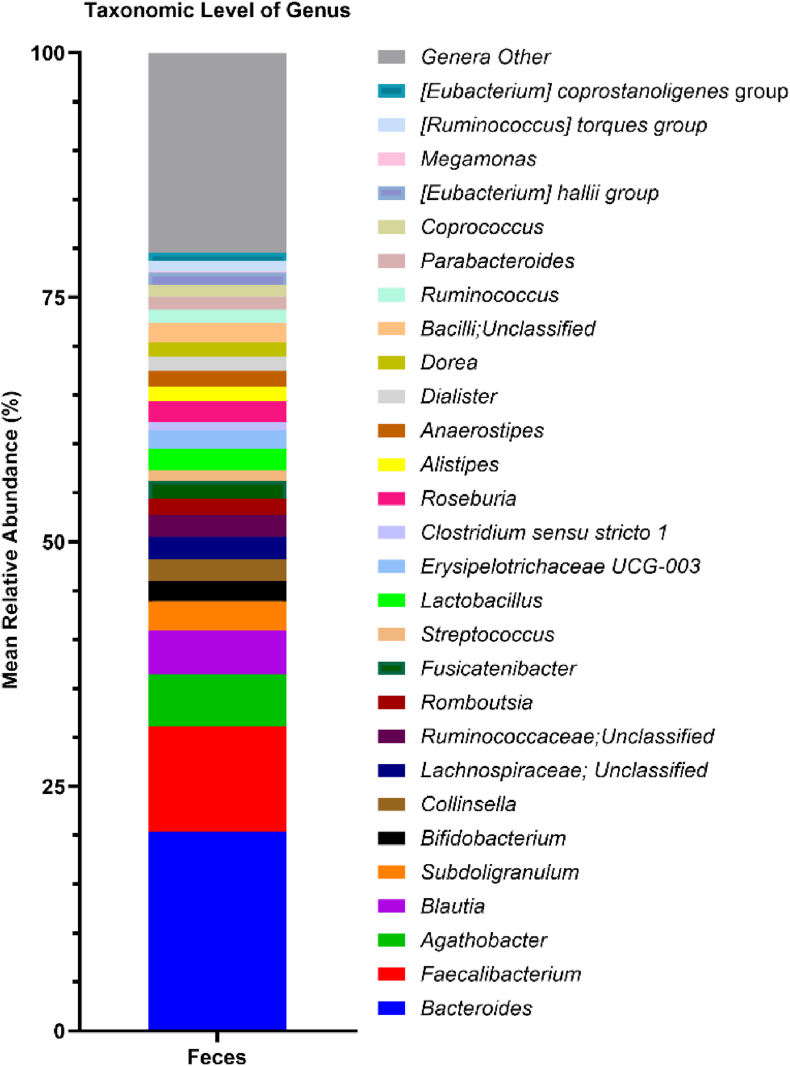
The percentage distribution and relative abundance (>1%) of fecal bacterial genera across samples.

In a smaller subset of 16 participants, stool samples were collected. Before performing correlations between fecal microbiota and immune fitness scores, fecal microbial profiles were evaluated at phylum (Fig. 3) and genus (Fig. 4) taxonomic levels to characterize the bacterial compositions of fecal samples from the participants.

Table A4 summarizes the correlations of alpha-diversity metrics and the F/B ratio with immune fitness scores. After bootstrapping, a significant negative Spearman's correlation was found between immune fitness scores and alpha-diversity index of Observed Features (r = −0.661). Thus, improved immune fitness corresponded to reduced microbial richness (see Fig. 4). In the context of immune fitness, the overall microbial profile suggests a community enriched in taxa known to contribute to immune regulation and mucosal homeostasis. The core microbiome of the participants consisted predominately of highly abundant short-chain fatty acids (SCFA) acetate- and butyrate-producing genera, like *Faecalibacterium*, *Agathobacter*, *Blautia*, *Subdoligranulum*, and *Bifidobacterium*, which are recognized contributors to colonic epithelial integrity, immune modulation, and maintenance of intestinal homeostasis. The relationship between these fecal genus-level abundances and immune fitness scores are summarized in Table A5.

A significant positive correlation was found between immune fitness and the relative abundance of putative anti-inflammatory SCFA butyrate-producing bacteria, *Lachnoclostridium* (r = 0.513; *p* = 0.042; BCa 95% CI_B_ = 0.002, 0.795). Significant negative correlations were found between immune fitness and the relative abundances of commensal gut bacterial genera *Colidextribacter* (r = −0.582; *p* = 0.018; BCa 95% CI_B_ = −0.845, −0.120) and *Lachnospiraceae FCS020* group (r = −0.504; *p* = 0.047; BCa 95% CI_B_ = −0.847, −0.016).

## 4 Discussion

**Fig. 5:**
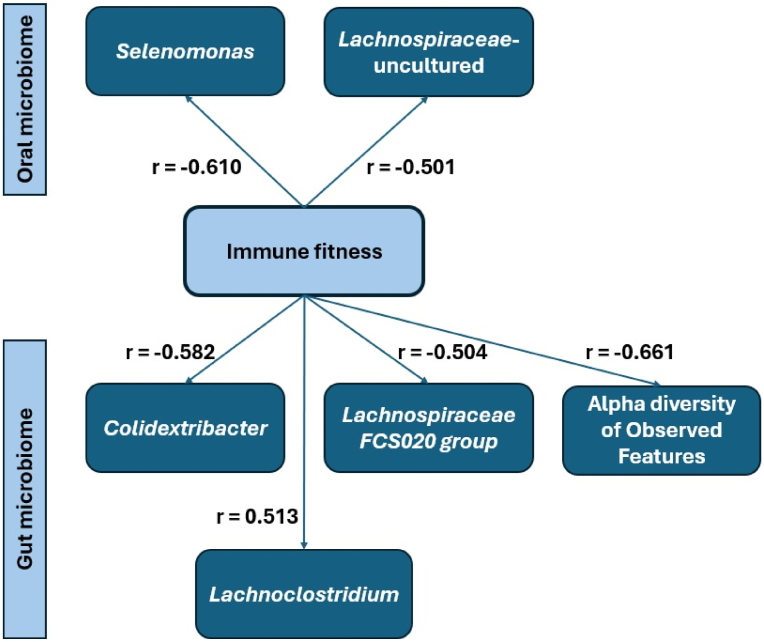
Significant relationships between immune fitness and oral and gut microbiota.

In this study, several significant associations, summarized in Fig. 5, were found between immune fitness and oral and gut microbiome compositions.

The salivary microbiome had statistically significant negative correlations between immune fitness scores and the relative abundances of genera *Selenomonas* (Cho et al., 2023) and *Lachnospiraceae* uncultured (Kim et al., 2022), whose bacterial abundances have been previously associated with human health and disease. These findings were further supported by salivary inflammation biomarker levels showing significant negative correlations between the salivary IL-1β and IL-8 concentrations and the relative abundance of putative anti-inflammatory genus *Lautropia* (Al-Kamel et al., 2019; Wirth et al., 2022), and a non-significant positive correlation between the salivary IL-1β concentration and the putative oral proinflammatory genus *Saccharimonadales* (formerly known as *TM7* or *Candidatus Saccharibacteria*) was found (Curtis et al., 2020; Esparbès et al., 2021; Qi et al., 2021b).

The fecal microbiome had a significant negative correlation between immune fitness scores and the alpha-diversity index of Observed Features, that represents the total number of unique genera detected in a sample, as well as how many different types of microbes are present within the sample (Cassol et al., 2025). Thus, better immune fitness scores were associated with this lower alpha-diversity metric, corresponding to reduced microbial richness (see Fig. 4), and potentially indicating a decrease in putative pathogenic bacteria and a more stable core microbiome composed of beneficial SCFA-producing bacteria. Additionally, the fecal samples of participants showed a significant positive correlation between immune fitness scores and the relative abundances of putative anti-inflammatory genus *Lachnoclostridium* (Liu et al., 2019), and significant negative correlations were found between immune fitness and the relative abundance of gut commensal bacteria *Colidextribacter* and the *Lachnospiraceae FCS020* group.

Previous research revealed significant correlations between self-reported immune fitness scores and salivary immune biomarker concentrations (Išerić et al., 2025a). Therefore, it was expected that both the salivary and fecal microbial abundances would be involved in immune function, reflected by significant correlations with self-reported immune fitness. Indeed, in line with our findings, previous research found that a high salivary genus-level abundance of *Selenomonas* was associated with experiencing various autoimmune and/or inflammatory diseases, including rheumatoid arthritis and periodontitis (Gao et al., 2022; Hespell et al., 2006; Faveri et al., 2008). Therefore, a higher relative abundance of *Selenomonas* in the salivary microbial profile could indicate that the body is facing an immune threat, leading to a lower perceived immune fitness.

The negative correlation between immune fitness and relative abundance of salivary *Lachnospiraceae* is in line with previous observations. While many members of the family *Lachnospiraceae* are commensal and butyrate producers, their presence in the oral cavity with the assigned unclassified label, has been associated with dysbiosis and inflammation, like periodontitis and oral infections (Benjamin et al., 2023). These data suggest that higher immune fitness scores are associated with a reduced relative abundance of potentially pathogenic or pro-inflammatory bacteria in saliva.

Oral *Lautropia* is commensal and does not pose a threat to human health, except when the host's immune system is suboptimal (Liu et al., 2024; Calheiros Cruz et al., 2022). For example, in patients with IBD the relative abundance of bacteria from the genus *Lautropia* was negatively correlated to various inflammatory biomarkers, including erythrocyte sedimentation rate and white blood cell count (Qi et al., 2021c). Furthermore, a higher relative abundance of oral *Lautropia* abundance was measured in people with good periodontal health, compared to patients with periodontitis (Papapanou et al., 2020; Colombo et al., 2009). These reports are in line with the negative correlation between oral *Lautropia* and salivary immune biomarker concentrations found in our study.

The observed positive correlation between immune fitness scores and gut genus *Lachnoclostridium* is also in line with previous research. *Lachnoclostridium* is an SCFA-producing bacterium, exerting its anti-inflammatory properties by producing butyrate (Markey et al., 2020). For example, higher levels of butyrate have been associated with better gut barrier function, thereby contributing to general health (Leibovitzh et al., 2022). Other research demonstrated that butyrate inhibits pro-inflammatory cytokine production, including IL-1β, IL-6 and IL-8, in human fetal small intestinal epithelial cells (Huang et al., 2022).

*Colidextribacter* is a commensal bacteria which also possesses SCFA-producing abilities, which are associated with anti-inflammatory effects (Wang et al., 2022). Therefore, the negative direction of the correlation between gut *Colidextribacter* abundance and immune fitness was somewhat unexpected. However, other studies also found negative associations between the relative abundance of *Colidextribacter* and immune-related health outcomes. For example, a study in mice found that mice with diabetes type 2, have a higher relative abundance of *Colidextribacter* compared to mice without diabetes (Wang et al., 2023). A different study conducted in healthy relatives of patients with Crohn's disease revealed a positive correlation between gut *Colidextribacter* abundance and increased gut permeability (Leibovitzh et al., 2022). These findings support the observed negative correlation between immune fitness and gut *Colidextribacter* abundance.

Finally, a negative correlation was found between immune fitness and relative abundance of the *Lachnospiraceae FCS020 group*. The negative direction of the observed association was unexpected, since the *Lachnospiraceae FCS020 group* are SCFA-producing species (O'Riordan et al., 2022). However, in mice with atherosclerosis, a greater *Lachnospiraceae FCS020 group* abundance has been associated with the production of various pro-inflammatory cytokines (Sun et al., 2021). Hence, the role of the *Lachnospiraceae FCS020 group* in immune function requires further study.

Taken together, our study showed that self-reported immune fitness is associated with the relative abundance of oral and gut microbiota implicated in immune function. While direct taxonomic comparisons between saliva and gut microbiota were not performed in this study, examining their associations with immune fitness in parallel revealed site-specific patterns without implying compositional equivalence. Strengths of the study included the use of both gut and oral microbiota to examine their associations with immune fitness, which was measured by a validated and reliable single-item scale (Verster et al., 2022). Limitations of the study comprises of its small sample size; however, this was addressed by applying bootstrapping and utilizing stringent criteria for correlations to be considered both statistically and biologically relevant. Nevertheless, due to the small sample size, no sex-based analyses could be performed. Given that both immune system functionality and microbiota composition are influenced by sex-specific biological factors, this limitation should be considered when interpreting our findings and in the design of future studies. Second, while short-read 16S rRNA sequencing offers broad taxonomic coverage, it lacks full species-level resolution and cannot directly infer microbial functional genes or pathways. Future studies using full-length 16S sequencing, metagenomics, or metabolomics could provide deeper insights into microbial activity and host–microbe interactions. Furthermore, participants of the study were thoroughly screened by the study physician and included if they were healthy and between 18 and 30 years old (Mackus et al., 2023). As such, the samples had a relatively narrow age range. Therefore, future studies should verify our findings in other age groups and different populations (e.g., patients with a diagnosed underlying disease). In these studies, the possible impact of lifestyle factors that may influence immune fitness (e.g., physical activity, daily diet) (Verster et al., 2022, 2023a; Išerić et al., 2026) should also be assessed. Finally, this study focused on simple correlations, although in fact, microorganisms interact collectively to impact immune fitness. Studies with a larger sample size will allow for more complex analyses and modeling of how the combined effects of different taxa impact immune fitness.

In conclusion, self-reported immune fitness is associated with the relative abundance of oral and gut microbiota that are involved in immune function.

## CRediT authorship contribution statement

**Guusje A. Ulijn:** Writing – review & editing, Writing – original draft, Conceptualization. **Emina Išerić:** Writing – review & editing, Conceptualization. **Aurora J.A.E. van de Loo:** Writing – review & editing, Investigation, Conceptualization. **Johan Garssen:** Writing – review & editing, Conceptualization. **Phillip A. Engen:** Writing – review & editing, Writing – original draft, Formal analysis, Data curation. **Ankur Naqib:** Writing – review & editing, Formal analysis, Data curation. **Stefan J. Green:** Writing – review & editing, Formal analysis, Data curation. **Ali Keshavarzian:** Writing – review & editing, Conceptualization. **Joris C. Verster:** Writing – review & editing, Writing – original draft, Formal analysis, Data curation, Conceptualization.

## Informed consent statement

Written informed consent was obtained from all participants.

## Institutional review board statement

The University of Groningen Psychology Ethics Committee approved the study (protocol code: ppo-015–002, approval date: 3 September 2015).

## Funding

These microbiome analyses were departmentally funded by 10.13039/501100001829Utrecht University and the Rush Center for Integrated Microbiome and Chronobiology Research at 10.13039/100008616Rush University Medical Center.

## Declaration of competing interest

Over the past 36 months, J.V. has received research grants from Danone and Inbiose and has acted as a consultant/expert advisor to KNMP, Komoinvest, Med Solutions, Mozand, Red Bull, and Sen-Jam Pharmaceutical. J.V. and E.I. have received travel support from Sen-Jam Pharmaceutical. J.V. owns stock from Sen-Jam Pharmaceutical. J.G. is part-time employee of Nutricia Research and received research grants from Nutricia research foundation, Top Institute Pharma, Top Institute Food and Nutrition, GSK, STW, NWO, Friesland Campina, CCC, Raak-Pro, and EU. The other authors have nothing to declare.

## Figures and Tables

**Table A1:** Relationship of immune fitness and salivary biomarkers with alpha diversity.

	Shannon's index	Simpson's index	Observed Features	Pielou's Evenness	F/B-ratio
**Immune fitness**	r = −0.229	r = −0.178	r = −0.112	r = −0.169	r = 0.05
	p = 0.232	p = 0.355	p = 0.562	p = 0.381	p = 0.769
	L = −0.577	L = −0.573	L = −0.524	L = −0.559	L = −0.292
	U = 0.164	U = 0.272	U = 0.324	U = 0.283	U = 0.390
**IL-1β**	r = −0.028	r = 0.161	r = −0.086	r = 0.076	r = −0.317
	p = 0.888	p = 0.412	p = 0.665	p = 0.700	p = 0.140
	L = −0.473	L = −0.318	L = −0.507	L = −0.400	L = −0.655
	U = 0.395	U = 0.559	U = 0.368	U = 0.511	U = 0.119
**IL-6**	r = −0.377	r = −0.302	r = −0.254	r = −0.359	r = −0.167
	p = 0.048	p = 0.118	p = 0.192	p = 0.061	p = 0.448
	L = −0.736	L = −0.682	L = −0.620	L = −0.25	L = −0.249
	U = 0.073	U = 0.130	U = 0.198	U = 0.077	U = 0.528
**IL-8**	r = −0.066	r = 0.093	r = −0.027	r = −0. 080	r = −0.255
	p = 0.753	p = 0.658	p = 0.897	p = 0.705	p = 0.239
	L = −0.496	L = −0.326	L = −0.516	L = −0.438	L = −0.616
	U = 0.349	U = 0.483	U = 0.408	U = 0.299	U = 0.151
**TNF-α**	r = −0.331	r = −0.185	r = −0.150	r = −0.263	r = −0.115
	p = 0.079	p = 0.337	p = 0.438	p = 0.168	p = 0.600
	L = −0.659	L = −0.549	L = −0.495	L = −0.625	L = −0.330
	U = 0.069	U = 0.224	U = 0.216	U = 0.152	U = 0.517

Abbreviations: F/B-ratio = Firmicutes-to-Bacteroidetes ratio, IL = interleukin, TNF = tumor necrosis factor, r = Spearman's correlation, p = p-value, L = lower limit of the bootstrapping confidence interval BCa 95% CI_B_, U = upper limit of the bootstrapping confidence interval BCa 95% CI_B_. None of the correlations was statistically significant.

**Table A2:** Correlations between immune fitness and genus abundance.

Saliva microbiome	Correlations with immune fitness		
Genus Taxonomic Level	r	p-value	BCa 95% CI_B_
*Corynebacterium*	−0.091	0.637	−0.505, 0.341
*Actinomyces*	−0.053	0.784	−0.397, 0.302
*Rothia*	0.062	0.750	−0.315, 0.433
*Atopobium*	−0.221	0.250	−0.633, 0.237
*Prevotella*	−0.237	0.216	−0.584, 0.162
*Alloprevotella*	0.205	0.286	−0.175, 0.554
*Porphyromonas*	0.011	0.953	−0.379, 0.401
*Capnocytophaga*	0.063	0.746	−0.368, 0.472
*Bergeyella*	0.121	0.533	−0.301, 0.511
*Campylobacter*	−0.293	0.122	−0.622, 0.123
*Streptococcus*	0.098	0.613	−0.323, 0.468
*Veillonella*	−0.047	0.810	−0.478, 0.388
*Granulicatella*	−0.109	0.575	−0.477, 0.265
*Gemella*	−0.024	0.900	−0.376, 0.331
*Megasphaera*	−0.237	0.216	−0.553, 0.136
***Selenomonas***	**−0.610**	**< 0.001**	**−0.832, -0.240**
*Oribacterium*	0.193	0.315	−0.160, 0.513
*Solobacterium*	−0.175	0.363	−0.558, 0.259
*Lachnoanaerobaculum*	−0.287	0.131	−0.650, 0.144
*Stomatobaculum*	−0.036	0.854	−0.402, 0.358
*Clostridia UCG-014*	−0.148	0.444	−0.446, 0.188
***Lachnospiraceae* uncultured**	**−0.501**	**0.006**	**−0.753, -0.145**
*Abiotrophia*	−0.092	0.635	−0.446, 0.289
*Peptostreptococcus*	0.055	0.778	−0.281, 0.379
*Mogibacterium*	−0.118	0.542	−0.493, 0.271
*Parvimonas*	0.218	0.256	−0.162, 0.555
*Dialister*	−0.131	0.499	−0.501, 0.310
*Eubacterium nodatum* group	−0.118	0.541	−0.487, 0.298
Selenomonadaceae unclassified	−0.265	0.164	−0.587, 0.106
*Catonella*	0.166	0.391	−0.192, 0.493
*Fusobacterium*	0.270	0.157	−0.136, 0.637
*Leptotrichia*	−0.332	0.078	−0.661, 0.079
*Saccharimonadales*	−0.228	0.235	−0.590, 0.189
*Absconditabacteriales (SR1)*	0.102	0.598	−0.279, 0.469
*TM7x*	0.026	0.892	−0.339, 0.394
*Pasteurellaceae* unclassified	0.252	0.188	−0.143, 0.581
*Neisseria*	0.117	0.545	−0.266, 0.474
*Lautropia*	0.070	0.717	−0.333, 0.455
*Haemophilus*	0.027	0.890	−0.388, 0.442
*Neisseriaceae* unclassified	0.027	0.890	−0.366, 0.422
*Aggregatibacter*	−0.095	0.622	−0.485, 0.304
*Cardiobacterium*	−0.009	0.964	−0.445, 0.428
*Treponema*	0.240	0.210	−0.144, 0.603

Spearman's correlations were considered statistically significant if each of the following 3 criteria was met: (a) the r-value was greater than ±0.5, (b) the p-value was smaller than 0.05, and (c) the BCa 95% CI_B_ bootstrapping interval did not contain the value zero.

**Table A3:** Correlations between cytokine concentrations and genus abundance.

Saliva microbiome	Correlations with IL-1β			Correlations with IL-8			Correlations with IL-6			Correlations with TNF-α		
Genus Taxonomic Level	r	p-value	BCa 95% CI_B_	r	p-value	BCa 95% CI_B_	r	p-value	BCa 95% CI_B_	r	p-value	BCa 95% CI_B_
*Corynebacterium*	−0.158	0.423	−0.562, 0.310	−0.140	0.506	−0.562, 0.318	−0.190	0.334	−0.490, 0.179	−0.028	0.885	−0.372, 0.324
*Actinomyces*	−0.114	0.562	−0.486, 0.253	−0.040	0.851	−0.476, 0.387	−0.045	0.822	−0.362, 0.293	−0.164	0.394	−0.482, 0.196
*Rothia*	0.027	0.890	−0.375, 0.410	0.004	0.984	−0.388, 0.389	0.123	0.533	−0.284, 0.489	0.331	0.079	−0.093, 0.698
*Atopobium*	0.029	0.833	−0.318, 0.382	0.174	0.406	−0.246, 0.573	0.210	0.283	−0.159, 0.537	−0.073	0.706	−0.449, 0.303
*Prevotella*	0.059	0.767	−0.315, 0.408	0.230	0.269	−0.192, 0.632	0.221	0.258	−0.128, 0.530	−0.082	0.673	−0.459, 0.302
*Alloprevotella*	−0.019	0.923	−0.428, 0.375	−0.079	0.708	−0.429, 0.298	−0.386	0.042	−0.698, −0.008	−0.361	0.054	−0.629, −0.014
*Porphyromonas*	0.078	0.692	−0.340, 0.457	−0.155	0.459	−0.539, 0.247	−0.334	0.083	−0.701, 0.117	−0.047	0.808	−0.420, 0.347
*Capnocytophaga*	−0.034	0.864	−0.409, 0.353	−0.217	0.297	−0.642, 0.232	−0.268	0.168	−0.638, 0.163	−0.066	0.733	−0.427, 0.315
*Bergeyella*	−0.109	0.581	−0.473, 0.279	−0.207	0.322	−0.613, 0.216	−0.408	0.031	−0.684, −0.041	−0.081	0.678	−0.407, 0.273
*Campylobacter*	0.139	0.480	−0.226, 0.461	0.212	0.310	−0.187, 0.566	0.066	0.738	−0.316, 0.420	−0.055	0.776	−0.404, 0.320
*Streptococcus*	−0.127	0.520	−0.492, 0.262	0.023	0.911	−0.368, 0.388	0.074	0.710	−0.326, 0.429	0.133	0.491	−0.263, 0.512
*Veillonella*	−0.039	0.844	−0.440, 0.360	0.088	0.674	−0.331, 0.507	0.167	0.396	−0.208, 0.507	−0.061	0.752	−0.448, 0.315
*Granulicatella*	0.308	0.111	−0.074, 0.612	0.122	0.560	−0.272, 0.491	0.160	0.416	−0.208, 0.500	0.434	0.019	0.103, 0.703
*Gemella*	−0.093	0.638	−0.439, 0.285	−0.030	0.887	−0.528, 0.416	−0.207	0.290	−0.545, 0.160	0.010	0.960	−0.449, 0.425
*Megasphaera*	−0.032	0.873	−0.383, 0.304	0.110	0.599	−0.273, 0.469	0.203	0.301	−0.160, 0.506	−0.081	0.676	−0.419, 0.283
*Selenomonas*	0.134	0.496	−0.298, 0.542	0.125	0.552	−0.294, 0.505	0.179	0.363	−0.207, 0.520	−0.025	0.896	−0.370, 0.318
*Oribacterium*	−0.354	0.065	−0.695, 0.085	−0.156	0.456	−0.485, 0.223	−0.309	0.110	−0.644, 0.111	−0.375	0.045	−0.665, 0.009
*Solobacterium*	0.134	0.496	−0.270, 0.476	0.025	0.904	−0.448, 0.480	−0.078	0.693	−0.368, 0.247	−0.064	0.742	−0.423, 0.325
*Lachnoanaerobaculum*	0.051	0.795	−0.380, 0.490	0.258	0.214	−0.173, 0.640	0.185	0.345	−0.211, 0.539	−0.060	0.758	−0.431, 0.344
*Stomatobaculum*	−0.218	0.265	−0.611, 0.215	−0.028	0.895	−0.427, 0.379	−0.093	0.637	−0.463, 0.302	−0.279	0.142	−0.593, 0.111
*Clostridia UCG-014*	0.086	0.664	−0.330, 0.459	−0.024	0.910	−0.453, 0.409	−0.187	0.341	−0.545, 0.237	−0.165	0.392	−0.539, 0.239
*Lachnospiraceae* uncultured	0.115	0.560	−0.334, 0.543	−0.042	0.842	−0.470, 0.379	−0.056	0.779	−0.503, 0.381	−0.205	0.286	−0.554, 0.191
*Abiotrophia*	−0.068	0.731	−0.472, 0.348	−0.201	0.336	−0.658, 0.305	−0.024	0.902	−0.400, 0.372	0.057	0.769	−0.315, 0.402
*Peptostreptococcus*	0.041	0.836	−0.317, 0.406	−0.159	0.447	−0.549, 0.257	−0.353	0.065	−0.641, 0.036	−0.094	0.626	−0.463, 0.292
*Mogibacterium*	0.058	0.769	−0.361, 0.456	−0.045	0.832	−0.461, 0.409	0.141	0.475	−0.250, 0.508	0.006	0.976	−0.412, 0.421
*Parvimonas*	0.030	0.879	−0.367, 0.394	0.136	0.517	−0.267, 0.492	−0.140	0.478	−0.465, 0.222	−0.072	0.711	−0.452, 0.299
*Dialister*	−0.087	0.659	−0.483, 0.308	0.206	0.323	−0.262, 0.614	−0.105	0.594	−0.438, 0.263	−0.190	0.324	−0.484, 0.135
*Eubacterium nodatum* group	0.280	0.148	−0.135, 0.605	0.103	0.624	−0.289, 0.472	−0.094	0.636	−0.434, 0.272	−0.044	0.819	−0.413, 0.320
Selenomonadaceae unclassified	−0.035	0.858	−0.431, 0.387	−0.041	0.845	−0.465, 0.356	−0.108	0.583	−0.470, 0.265	−0.198	0.304	−0.544, 0.192
*Catonella*	0.151	0.445	−0.261, 0.507	0.212	0.309	−0.258, 0.628	−0.168	0.393	−0.520, 0.228	−0.130	0.503	−0.488, 0.274
*Fusobacterium*	−0.043	0.827	−0.444, 0.344	0.023	0.931	−0.392, 0.436	−0.325	0.092	−0.660, 0.078	−0.216	0.260	−0.574, 0.227
*Leptotrichia*	0.048	0.810	−0.403, 0.460	−0.022	0.916	−0.447, 0.419	−0.173	0.379	−0.593, 0.293	−0.132	0.494	−0.562, 0.337
*Saccharimonadales*	0.409	0.031	0.053, 0.686	0.296	0.150	−0.101, 0.606	0.089	0.652	−0.260, 0.411	0.127	0.512	−0.221, 0.464
*Absconditabacteriales* (*SR1*)	−0.032	0.871	−0.422, 0.372	−0.224	0.282	−0.651, 0.211	−0.216	0.269	−0.622, 0.256	−0.132	0.493	−0.516, 0.269
*TM7x*	0.146	0.458	−0.250, 0.497	−0.045	0.829	−0.419, 0.337	−0.201	0.304	−0.497, 0.138	−0.192	0.318	−0.573, 0.235
*Pasteurellaceae* unclassified	−0.226	0.249	−0.577, 0.207	−0.435	0.030	−0.720, −0.047	−0.201	0.304	−0.582, 0.244	−0.049	0.803	−0.460, 0.387
*Neisseria*	0.053	0.788	−0.290, 0.395	0.028	0.894	−0.408, 0.476	−0.189	0.336	−0.575, 0.279	0.068	0.728	−0.296, 0.433
*Lautropia*	**−0.591**	**< 0.001**	**−0.853, -0.189**	**−0.546**	**0.005**	**−0.845, -0.078**	−0.427	0.024	−0.723, −0.040	−0.411	0.027	−0.716, −0.036
*Haemophilus*	−0.263	0.176	−0.569, 0.106	−0.180	0.389	−0.549, 0.214	−0.192	0.328	−0.528, 0.190	−0.090	0.643	−0.458, 0.302
*Neisseriaceae* unclassified	0.063	0.748	−0.339, 0.467	0.021	0.920	−0.479, 0.506	0.060	0.762	−0.305, 0.410	0.150	0.438	−0.212, 0.457
*Aggregatibacter*	−0.304	0.116	−0.664, 0.105	−0.219	0.292	−0.628, 0.266	−0.283	0.144	−0.601, 0.107	−0.279	0.143	−0.564, 0.080
*Cardiobacterium*	−0.330	0.086	−0.632, 0.052	−0.323	0.115	0.652, 0.076	−0.311	0.108	−0.635, 0.070	−0.128	0.509	−0.496, 0.254
*Treponema*	0.016	0.936	−0.351, 0.378	0.067	0.751	−0.361, 0.454	−0.197	0.316	−0.561, 0.198	−0.160	0.407	−0.480, 0.194

Spearman's correlations were considered statistically significant if each of the following 3 criteria was met: (a) the r-value was greater than 0.5, (b) the p-value was smaller than ±0.05, and (c) the BCa 95% CI_B_ bootstrapping interval did not contain the value zero.

**Table A4:** Relationship of immune fitness with gut alpha diversity and F/B-ratio.

	Shannon's index	Simpson's index	Observed Features	Pielou's Evenness	F/B-ratio
**Immune fitness**	r = −0.490	r = −0.340	r = −0.661	r = −0.150	r = 0.021
	p = 0.064	p = 0.216	p = 0.007	p = 0.593	p = 0.940
	L = −0.830	L = −0.743	L = −0.923	L = −0.687	L = −0.587
	U = 0.096	U = 0.304	U = −0.091	U = 0.443	U = 0.675

Correlations were considered statistically significant if each of the following 3 criteria was met: (a) the r-value was greater than ±0.5, (b) the p-value was smaller than 0.05, and (c) the BCa 95% CI_B_ bootstrapping interval did not contain the value zero. Abbreviations: F/B-ratio = *Firmicutes*-to-*Bacteroidetes* ratio, IL = interleukin, TNF = tumor necrosis factor, r = Spearman's correlation, p = p-value, L = lower limit of the bootstrapping confidence interval BCa 95% CI_B_, U = upper limit of the bootstrapping confidence interval BCa 95% CI_B_.

**Table A5:** Correlations between immune fitness and genus abundance in the gut.

Gut microbiome	Correlations with immune fitness		
Genus Taxonomic Level	r	p-value	BCa 95% CI_B_
*Bacteroides*	−0.096	0.724	−0.670, 0.527
*Faecalibacterium*	−0.054	0.841	−0.579, 0.532
*Agathobacter*	0.054	0.843	−0.515, 0.598
*Blautia*	−0.090	0.741	−0.455, 0.593
*Subdoligranulum*	−0.497	0.050	−0.834, 0.014
*Bifidobacterium*	−0.025	0.927	−0.521, 0.517
*Collinsella*	−0.185	0.493	−0.595, 0.280
*Lachnospiraceae*	−0.074	0.786	−0.584, 0.520
*Ruminococcaceae*	−0.452	0.079	−0.761, 0.009
*Romboutsia*	−0.259	0.332	−0.177, 0.598
*Fusicatenibacter*	−0.180	0.506	−0.667, 0.312
*Streptococcus*	−0.084	0.757	−0.643, 0.490
*Lactobacillus*	0.032	0.907	−0.579, 0.585
*Erysipelotrichaceae UCG-003*	0.134	0.620	−0.309, 0.580
*Clostridium sensu stricto 1*	−0.257	0.337	−0.666, 0.243
*Roseburia*	−0.081	0.766	−0.568, 0.423
*Alistipes*	−0.490	0.054	−0.904, 0.110
*Anaerostipes*	0.224	0.405	−0.372, 0.703
*Dialister*	−0.241	0.368	−0.710, 0.285
*Dorea*	−0.027	0.922	−0.576, 0.479
*Bacilli*	0.393	0.132	X
*Ruminocccus*	−0.015	0.956	−0.695, 0.635
*Parabacteroides*	−0.345	0.191	−0.810, 0.224
*Coprococcus*	−0.257	0.336	−0.714, 0.331
*Eubacterium hallii* group	−0.283	0.289	−0.766, 0.413
*Megamonas*	−0.218	0.417	−0.578, 0.139
*Ruminococcus torques* group	−0.124	0.648	−0.627, 0.460
*Eubacterium coprostanoligenes* group	−0.428	0.098	−0.793, 0.124
*NK4A214* group	−0.446	0.083	−0.783, 0.109
*UCG-002*	−0.510	0.044	−0.839, 0.047
*Prevotella*	−0.444	0.085	−0.773, 0.146
*Barnesiella*	−0.272	0.307	−0.800, 0.373
*Turicibacter*	0.179	0.508	−0.321, 0.574
*Christensenellaceae R-7* group	−0.385	0.141	−0.678, 0.070
*Lachnospira*	−0.560	0.024	−0.899, 0.032
*UCG-005*	−0.466	0.069	−0.826, 0.068
*Intestinibacter*	−0.214	0.426	−0.705, 0.441
*Butyricicoccus*	−0.412	0.112	−0.871, 0.183
*Phascolarctobacterium*	−0.055	0.839	−0.637, 0.508
*Megasphaera*	−0.259	0.332	−0.760, 0.368
*Sutterella*	−0.010	0.970	−0.534, 0.582
***Lachnoclostridium***	**0.513**	**0.042**	**0.002, 0.795**
*CAG-352*	0.283	0.287	−0.125, 0.627
*Eubacterium ventriosum* group	−0.161	0.553	−0.635, 0.360
*Prevotellaceae NK3B31* group	−0.309	0.245	X
*Alloprevotella*	−0.282	0.291	−0.638, 0.031
*Escherichia Shigella*	−0.330	0.211	−0.746, 0.224
*Tyzzerella*	−0.154	0.569	−0.684, 0.475
***Colidextribacter***	**−0.582**	**0.018**	**−0.845, -0.120**
*Eubacterium eligens* group	0.013	0.961	−0.531, 0.556
*CAG-56*	0.256	0.339	−0.280, 0.723
*Veillonella*	0.107	0.693	−0.430, 0.579
*Monoglobus*	−0.241	0.369	−0.753, 0.405
*Lachnospiraceae ND3007* group	−0.133	0.624	−0.569, 0.409
*Incertae Sedis*	−0.380	0.147	−0.887, 0.330
*Odoribacter*	−0.380	0.147	−0.798, 0.235
*Akkermansia*	0.106	0.696	−0.382, 0.570
*Paraprevotella*	−0.165	0.542	−0.699, 0.427
*Slackia*	0.051	0.852	−0.514, 0.626
*Lachnospiraceae NK4A136* group	−0.251	0.348	−0.761, 0.418
*Desulfovibrio*	0.036	0.894	−0.440, 0.460
*Clostridia UCG-014*	−0.325	0.219	−0.682, 0.094
*Ruminococcus gauvreauii* group	−0.386	0.140	−0.729, 0.090
*Eubacterium xylanophilum* group	−0.247	0.356	−0.735, 0.357
*Clostridium methylpentosum* group	−0.056	0.836	−0.420, 0.311
*Muribaculaceae*	−0.268	0.316	−0.750, 0.411
*Ruminococcus gnavus*_group	0.084	0.756	−0.437, 0.587
*Bilophila*	−0.162	0.548	−0.607, 0.327
*Lachnospiraceae UCG-004*	0.027	0.922	−0.493, 0.570
*Ruminococcaceae* uncultured	−0.392	0.133	−0.803, 0.181
*Adlercreutzia*	−0.121	0.655	−0.617, 0.411
*Terrisporobacter*	−0.401	0.124	−0.828, 0.218
*RF39*	−0.181	0.501	−0.529, 0.192
*Oscillospiraceae* uncultured	−0.239	0.372	−0.835, 0.436
*Erysipelatoclostridium*	−0.108	0.691	−0.610, 0.424
***Lachnospiraceae FCS020* group**	**−0.504**	**0.047**	**−0.847, -0.016**
*Lachnospiraceae UCG-001*	−0.305	0.251	−0.683, 0.174
*Eubacterium siraeum* group	−0.250	0.351	−0.649, 0.246
*Oscillibacter*	−0.066	0.807	−0.516, 0.394
*Lachnospiraceae UCG-010*	−0.078	0.774	−0.589, 0.481

Spearman's correlations were considered statistically significant if each of the following 3 criteria was met: (a) the r-value was greater than ±0.5, (b) the p-value was smaller than 0.05, and (c) the BCa 95% CI_B_ bootstrapping interval did not contain the value zero. X = no BCa 95% CI_B_ was calculated.

## Data Availability

Data will be made available on request.
